# Survey and analysis of the prevalence of tobacco use among patients with severe mental illness at a tertiary specialized psychiatric medical center in China

**DOI:** 10.3389/fpsyt.2025.1566356

**Published:** 2025-07-30

**Authors:** Hai-Bo Zheng, Chang-Qing Yu, Qiu-Ming Ji, Bing Liu, Yuan-Yuan Liu, Hai-Ying Chen, Yu-Lin Cao, Ji-Fen Gong, Ting Chen

**Affiliations:** ^1^ Department of Psychiatry, Wuhan Wudong Hospital, The Second Mental Hospital of Wuhan, Wuhan, Hubei, China; ^2^ School of Public Health, Medical School, Wuhan University of Science and Technology, Wuhan, Hubei, China

**Keywords:** severe mental illness, tobacco use, willingness to quit smoking, nicotine dependence, China

## Abstract

**Background:**

In China, the coexistence of mental illness and tobacco dependence is a major public health issue. With around 300 million smokers and over one million annual smoking-related deaths, the resulting social and economic burdens are considerable.

**Methods:**

A cross-sectional study design was employed to gather data on tobacco use, readiness to quit smoking, and nicotine dependence from a random sample of 738 patients diagnosed with severe mental illness. Data analysis, which included descriptive statistics and multivariate logistic regression analysis, was conducted via SPSS 27.0.

**Results:**

The findings revealed that the smoking prevalence among patients with severe mental illness was 52.03%, significantly higher than that of the general population. Multifactorial logistic regression analysis indicated that male gender(OR=10.041, 95% CI: 6.499-15.513), Han ethnicity(OR=3.263, 95% CI: 1.053-10.108), worse economic status (OR=2.540, 95% CI: 1.424-4.529), family history of smoking (OR=6.474, 95% CI: 4.211-9.952), outpatient status(OR=2.294, 95% CI: 1.433-3.674), family history of mental illness (OR=1.756, 95% CI: 1.129-2.731), history of drug exposure(OR=2.074, 95% CI: 1.244-3.458), and history of alcohol consumption(OR=5.216, 95% CI: 3.037-8.960) were independent risk factors for smoking in this patient group. Furthermore, there were significant differences in nicotine dependence levels across different psychiatric diagnoses, with patients diagnosed with schizophrenia and bipolar disorder exhibiting higher levels of nicotine dependence compared to those with paranoid disorder who showed lower levels.

**Conclusions:**

The study elucidates the complex nature and critical determinants of tobacco use patterns among individuals with severe mental illness, providing a solid scientific foundation for developing targeted intervention strategies.

**China Clinical Trial Registry registration number:**

ChiCTR2400088459

## 1 Introduction

In China, mental illness and tobacco dependence constitute a complex public health problem. According to epidemiological data, there are approximately 300 million smokers in China, accounting for nearly 40% of the world’s total tobacco consumption, and the resulting number of smoking-related deaths is the highest in the world, with more than one million people losing their lives each year (1, 2). This not only imposes a substantial economic burden on Chinese society but also poses a significant threat to public health and quality of life (3).

In the field of mental health, the World Health Organization’s 2022 report highlights a growing global burden of mental illness, particularly during the COVID-19 pandemic, which witnessed a significant increase in the incidence of mental disorders (4). The situation in China is similar, with the number of registered patients with severe mental illness (SMI) reaching 6.43 million by the end of 2020 (5). SMI refers to a category of severe mental disorders that arise from neurobiological abnormalities and are characterized by significant and persistent functional impairments. The core clinical features include severely impaired reality-testing abilities and/or substantial deficits in behavioral regulation, often accompanied by pronounced cognitive impairments. These conditions commonly lead to a marked deterioration or complete loss of socio-occupational functioning and are associated with high rates of relapse and long-term disability, thereby imposing a substantial burden on public health systems. In China, SMI specifically refers to the six major categories of mental illnesses that have been incorporated into the standardized management framework of the National Basic Public Health Service Program since 2009 (6): schizophrenia, schizoaffective disorder, paranoid disorder, bipolar disorder, epilepsy-related mental disorders, and intellectual disability with mental disorders (7).

In China, the prevalence of tobacco use among individuals with SMI is significantly greater than that in the general population (8); However, this issue has garnered insufficient attention. On the one hand, tobacco consumption exacerbates both the economic and physical health burdens faced by this demographic. On the other hand, smoking significantly affects the recurrence and prognosis of their conditions (9–11). These consequences not only compromise individual health outcomes, but also hinder the objectives of China’s “Healthy China 2030” strategy, which aims to reduce the national smoking rate to 20% by 2030 through comprehensive tobacco control initiatives.

Despite the extensive research on tobacco use both domestically and internationally, studies focusing specifically on patients with SMI, which primarily address schizophrenia and bipolar disorder, remain limited. Consequently, this study aims to employ a cross-sectional survey design to examine both outpatients and inpatients diagnosed with severe mental disorders at a tertiary mental health hospital in Wuhan. The objective of this to achieve a comprehensive understanding of the current status of tobacco use, smoking cessation intentions, and levels of nicotine dependence among individuals with SMIs in China. Additionally, this research sought to analyze the factors that characterize the high prevalence of smoking in this population to provide a theoretical basis for the cessation of smoking in this population.

## 2 Methods

### 2.1 Survey respondents

The sample for this survey comprised patients diagnosed with SMI, including both inpatients and outpatients, at a tertiary psychiatric specialty medical center in Wuhan, Hubei Province, China. Data collection occurred from September to November 2024.

### 2.2 Ethical considerations

The purpose and procedures of the study were explained to the participating patients prior to their commencement, and approval was obtained from the Ethics Committee of Wuhan Wudong Hospital. All participants provided written informed consent. Additionally, the study was registered with the China Clinical Trial Registry (ChiCTR) under the registration number ChiCTR2400088459.

### 2.3 Questionnaire design

For this study, we utilized a structured, self-developed questionnaire specifically designed to investigate tobacco use among individuals with SMI. The questionnaire comprises four main sections: (1) Informed consent. This section provides a detailed explanation of the survey objectives, the significance of the research, the nature of the investigation, the measures taken to ensure data confidentiality, and the comprehensive instructions for completing the questionnaire. (2) Demographic and Clinical Information. Adapted from the China Mental Health Survey (CMHS) (12), this section aims to collect essential sociodemographic and clinical data, including gender, age, educational attainment, ethnicity, marital status, economic status, psychiatric diagnosis, duration of illness, family history of mental disorders, and comorbid conditions. (3) Tobacco Use and Smoking Cessation Intention Assessment. This section draws upon the World Health Organization’s Global Adult Tobacco Survey (GATS) (13) framework and is primarily used to evaluate current smoking behaviors and cessation intentions among SMI patients, covering patterns of tobacco consumption and motivation toward quitting. (4) Nicotine Dependence Assessment. We incorporated the Fagerström test for nicotine dependence (FTND) to evaluate nicotine dependence levels in SMI patients. The FTND is a widely recognized tool for assessing nicotine dependence, with scores ranging from 0 to 10. Dependence levels are categorized as follows: 0–3 = low, 4–6 = moderate, and 7–10 = high. The Chinese version of the FTND has been validated for reliability in psychiatric populations (14). A pilot study was conducted prior to the formal implementation of the questionnaire to verify the content validity and ensure the efficacy of data collection.

### 2.4 Sample size calculation

Prior to initiating the research study, a preestimated sample size calculation was conducted, which indicated that a minimum of 511 valid questionnaires were necessary to ensure the reliability and validity of the findings. The sample size estimation was based on the following formula:


$$\text{N}=\pi_{0}\left(1-\pi_{0}\right){\left(\frac{\mu_{\alpha }+\mu_{\beta }}{\pi_{1}-\pi_{0}}\right)}^{2}$$

*π_0_*—— Tobacco use rates in patients with SMI reported in previous studies;

*π_1_*—— Preanalytically derived rates of tobacco use among patients with SMI.

Additionally, we calculated the Events per Predictor Variable (EPV) to assess model robustness. With 384 smoking events and 21 predictors in the final model, the EPV value of 18.3 exceeds the recommended threshold of EPV≥10, indicating sufficient statistical power for the regression analysis.

### 2.5 Inclusion and exclusion criteria

The inclusion criteria for participants were as follows: 1) met the diagnostic criteria for one of the six major categories of SMI (including schizophrenia, schizoaffective disorder, paranoid disorder, bipolar disorder, epilepsy-related mental disorders, and intellectual disability with mental disorders) as defined by the International Classification of Diseases, Eleventh Revision (ICD-11); 2) participants had to have a stable condition that allowed them to complete the Tobacco Use Survey questionnaire; and 3) they had to provide voluntary informed consent to participate in the current Tobacco Use Survey study.

The exclusion criteria were as follows: 1) individuals with communication disorders or severe intellectual disabilities that preclude effective participation in the tobacco use questionnaire; 2) individuals with significant physical illnesses(e.g., advanced cancer, severe cardiopulmonary dysfunction, or neurodegenerative diseases) that may compromise cognitive capacity or study participation; 3) individuals who declined to participate in the Tobacco Use Survey study.

### 2.6 Implementation and quality control of the survey organization

The survey adopts a two-stage cluster sampling design. In the first stage, inpatient and outpatient departments were designated as clusters, and a random selection of these clusters was conducted to identify the primary sampling units. In the second stage, for each selected primary unit, individual participants were randomly selected from the patient population within that unit using a random number table approach. Considering the unique characteristics of patients with mental illness, the survey employs an offline interview questionnaire method. Specifically, trained psychiatrists conducted one-on-one interviews with each participant and completed the questionnaire on their behalf on the basis of the responses provided. To ensure confidentiality, participants are assigned anonymous identification numbers. All the investigators, who are required to be qualified psychiatrists, undergo unified training to ensure proficiency in both survey methods and quality control procedures.

### 2.7 Data analysis

First, we quantified the current status of tobacco use and willingness to quit among diverse populations by specifically calculating the prevalence of tobacco use in patients with SMI across various demographic and clinical characteristics. Next, we examined the distribution of nicotine dependence levels among patients with different diagnoses of SMI. Finally, we constructed multifactor logistic regression models with backward elimination (*P*<0.05) to investigate associations between tobacco use and demographic/clinical characteristics, while assessing multicollinearity via variance inflation factors (VIFs < 5). All data management and analyses were performed via SPSS 27.0.

### 2.8 Reporting standards

In accordance with established guidelines for observational research, this study adhered rigorously to the Strengthening the Reporting of Observational Studies in Epidemiology (STROBE) checklist to ensure comprehensive and transparent reporting of both the methodology and results.

## 3 Results

A total of 900 patients were surveyed in this research study, with 822 agreeing to participate (91.33%). However, 84 participants failed to complete the questionnaire due to worsening mental symptoms, difficulties in comprehension, or withdrawal during the process. Ultimately, 738 valid questionnaires were collected(89.75% of participants), which included 525 inpatients and 213 outpatients.

### 3.1 Current status of tobacco use among patients with SMI

The age of the studied patients ranged from 18–81 years, with a mean age of 46.69 ± 13.71 years. Among the total sample of 738 patients, 384 (52.03%) were current smokers. The smoking prevalence was significantly greater among outpatients (65.73%) than among inpatients (46.48%). Additionally, 45 patients (6.10% of the total) had quit smoking, with 71.11% being male and 28.89% being female. Among the 384 current smokers, 137 (35.68%) expressed an intention to quit, comprising 115 males (83.94%) and 22 females (16.06%). The survey results indicated that the majority of smokers had more than 10 years of smoking history (43.49%), whereas the smallest proportion had less than one year of smoking history (5.73%). In terms of daily cigarette consumption, the largest group smoked between 21 and 30 cigarettes per day (37.76%), whereas the smallest group smoked ≤10 cigarettes per day (19.79%). The most common reason for smoking was stress or emotional relief (44.79%), whereas socializing was the least common reason (12.50%) (**Table 1**).

**Table 1:** Tobacco use among individuals with SMI (N = 384).

Variable	Category	Frequency (%)
Duration of smoking (years)		
	< 1	22(5.73)
	1-5	104(27.08)
	6-10	97(25.26)
	> 10	167(43.49)
Daily cigarette consumption (sticks)		
	≤ 10	76(19.79)
	11-20	102(26.56)
	21-30	145(37.76)
	≥ 31	61(15.89)
The initial motivation for smoking		
	Imitation	115(29.95)
	Socializing	48(12.50)
	Relieving stress or emotion	172(44.79)
	Others	49(12.76)

SMI, Severe Mental Illness.

### 3.2 Comparison of demographic and clinical characteristics between tobacco users and nonusers among patients with SMI

A total of 738 patients were divided into two groups: 384 in the smoking group and 354 in the nonsmoking group (including former smokers), and sociodemographic data and clinical characteristics were compared between the two groups. The results revealed statistically significant differences in tobacco use among patients with SMIs across various demographic and clinical factors, including gender, age, ethnicity, marital status, occupation type, economic status, family smoking history, patient origin, illness duration, family history of mental illness, history of drug exposure, alcohol use history, oral disease, pulmonary disease, and cerebral infarction or hemorrhage (**Tables 2**, **3**).

**Table 2:** Sociodemographic comparison between smoking and non-smoking patients with SMI.

Variable	Smoking group (n=384)	Non-smoking group (n=354)	*χ^2^*	*P-value*
Gender				
Male	309 (80.47)	87 (24.57)	231.407	<0.001
Female	75 (19.53)	267 (75.43)		
Age (years)				
18-39	158 (41.15)	104 (29.38)	13.867	<0.001
40-59	159 (41.41)	158 (44.63)		
≥60	67 (17.44)	92 (25.99)		
Ethnicity				
Han Chinese	377 (98.18)	335 (94.63)	6.808	0.009
Other ethnicity	7 (1.82)	19 (5.37)		
Education level				
Elementary school and below	58 (15.10)	43 (12.15)	1.396	0.706
Junior high/middle/high school	253 (65.88)	243 (68.64)		
College/Undergraduate	63 (16.41)	59 (16.67)		
Postgraduate and above	10 (2.61)	9 (2.54)		
Marital status				
Unmarried	214 (55.73)	158 (44.63)	13.782	0.001
Married	100 (26.04)	93 (26.27)		
Divorced/widowed	70 (18.23)	103 (29.10)		
Place of residence				
Rural	100 (26.04)	76 (21.47)	2.121	0.145
Urban	284 (73.96)	278 (78.53)		
Occupation				
Unemployed	234 (60.94)	197 (55.65)	14.127	<0.001
Employed	85 (22.13)	58 (16.38)		
Retired	65 (16.93)	99 (27.97)		
Insurance				
Employee health insurance	141 (36.72)	143 (40.40)	1.111	0.574
Resident medical insurance	207 (53.91)	178 (50.28)		
Self-financed	36 (9.37)	33 (9.32)		
Economic situation				
Poor	126 (32.81)	77 (21.75)	11.302	<0.001
Well	258 (67.19)	277 (78.25)		
Family smoking exposure				
No	125 (32.55)	269 (75.99)	139.652	<0.001
Yes	259 (67.45)	85 (24.01)		

Poor indicates worse economic status; The sum of percentages may not equal 100% due to rounding.

SMI, Severe Mental Illness; χ², Chi-Square Test.

**Table 3:** Comparison of clinical characteristics of smoking and non-smoking groups of patients with SMI.

Variable	Smoking group (n=384)	Non-smoking group (n=354)	*χ^2^*	*P-value*
Source of patients				
Outpatients	140 (36.46)	73 (20.62)	22.5	<0.001
Inpatients	244 (63.54)	281 (79.38)		
Type of mental illness				
Schizophrenia	217 (56.51)	214 (60.45)	5.577	0.35
Bipolar disorder	122 (31.77)	99 (27.97)		
Schizoaffective disorder	11 (2.86)	8 (2.26)		
Paranoid disorder	4 (1.04)	7 (1.98)		
Epilepsy-related mental disorders	8 (2.08)	9 (2.54)		
Intellectual disability with mental disorders	22 (5.74)	17 (4.80)		
Duration of illness (years)				
<1	43 (11.20)	33 (9.32)	13.895	0.003
1-5	53 (13.80)	30 (8.47)		
6-10	67 (17.45)	42 (11.86)		
>10	221 (57.55)	249 (70.35)		
First-episode psychosis				
No	347 (90.36)	333 (94.07)	3.488	0.062
Yes	37 (9.64)	21 (5.93)		
Family history of mental illness				
No	216 (56.25)	247 (69.77)	14.411	<0.001
Yes	168 (43.75)	107 (30.23)		
History of substance use				
No	266 (69.27)	301 (85.03)	25.691	<0.001
Yes	118 (30.73)	53 (14.97)		
Alcohol use history				
No	153 (39.85)	35 (9.89)	87.068	<0.001
Yes	231 (60.15)	319 (90.11)		
Oral health problems				
No	233 (60.68)	189 (53.39)	3.995	0.046
Yes	151 (39.32)	165 (46.61)		
Hypertension				
No	154 (40.10)	127 (35.88)	1.397	0.237
Yes	230 (59.90)	227 (64.12)		
Diabetes				
No	86 (22.40)	84 (23.73)	0.185	0.667
Yes	298 (77.60)	270 (76.27)		
Dyslipidemia				
No	110 (28.65)	96 (27.12)	0.213	0.644
Yes	274 (71.35)	258 (72.88)		
Lung disease				
No	229 (59.64)	270 (76.27)	23.28	<0.001
Yes	155 (40.36)	84 (23.73)		
Cerebrovascular disease				
No	89 (23.18)	117 (33.05)	8.924	0.003
Yes	295 (76.82)	237 (66.95)		

The sum of percentages may not equal 100% due to rounding.

SMI, Severe Mental Illness; χ², Chi-Square Test.

### 3.3 Analysis of factors influencing tobacco use among patients with SMI

A multivariate logistic regression analysis was conducted using the aforementioned 15 factors as independent variables and smoking status as the dependent variable. The results indicated that being male (OR=10.041, 95% CI: 6.499-15.513), Han Chinese ethnicity (OR=3.263, 95% CI: 1.053-10.108), worse economic status (OR=2.540, 95% CI: 1.424-4.529), having family members who smoked (OR=6.474, 95% CI: 4.211-9.952), an outpatient status (OR=2.294, 95% CI: 1.433-3.674), a positive family history of psychiatric disorders (OR=1.756, 95% CI: 1.129-2.731), a history of drug exposure (OR=2.074, 95% CI: 1.244-3.458), and a history of alcohol consumption (OR=5.216, 95% CI: 3.037-8.960) were significant independent risk factors for smoking in patients with schizophrenia (all P<0.05) (**Table 4**). All retained variables exhibited VIFs between 1.2 and 3.8, indicating no significant multicollinearity in the final model.

**Table 4:** Analysis of factors associated with tobacco use among patients with SMI.

Variable	*b*	*SE*	*Wald*	*OR*	95%*CI*	*P-value*
Male	2.307	0.222	108.003	10.041	6.499~15.513	<0.001
Han Chinese	1.183	0.577	4.204	3.263	1.053~10.108	0.04
Worse economic status	0.932	0.295	9.976	2.540	1.424~4.529	0.002
Household smoking exposure	1.868	0.219	72.468	6.474	4.211~9.952	<0.001
Outpatients	0.83	0.24	11.951	2.294	1.433~3.674	<0.001
Positive family history of mental illness	0.563	0.225	6.247	1.756	1.129~2.731	0.012
Substance use	0.73	0.261	7.828	2.074	1.244~3.458	0.005
Alcohol use	1.652	0.276	35.805	5.216	3.037~8.960	<0.001
Comorbid oral disease	0.298	0.219	1.857	1.347	0.878~2.069	0.173
Comorbid lung disease	0.166	0.233	0.506	1.181	0.747~1.865	0.477
Comorbid cerebrovascular disease	0.309	0.284	1.185	1.363	0.781~2.378	0.276
Age (years)^a^						
40-59	0.236	0.289	0.664	1.266	0.193~1.779	0.415
≥60	0.257	0.402	0.409	1.293	0.240~1.362	0.523
Marital status^b^						
Unmarried	0.1	0.264	0.145	1.106	0.659~1.855	0.704
Divorced/widowed	-0.089	0.327	0.075	0.914	0.482~1.735	0.784
Occupation^c^						
Unemployed	0.063	0.345	0.033	1.065	0.542~2.092	0.856
Employed	0.031	0.383	0.007	1.032	0.487~2.186	0.935
Disease duration (years)^d^						
1-5	0.703	0.492	2.035	2.019	0.769~5.301	0.154
6-10	0.033	0.451	0.005	1.034	0.427~2.502	0.942
>10	-0.12	0.401	0.09	0.887	0.404~1.945	0.764

Model fit: Hosmer-Lemeshow *P* = 0.756.

^a^refers to 18–39 years of age; ^b^refers to married; ^c^refers to retired; and ^d^refers to <1 year as the reference variable.

b, Regression coefficient; SE, Standard Error; Wald, Wald Test; OR, Odds Ratio; CI, Confidence Interval.

### 3.4 Tobacco dependence among patients with various SMIs

Among the 384 patients with smoking-related psychosis, nicotine dependence levels were distributed as follows: high and very high dependence accounted for 51.61% of patients with schizophrenia and 49.18% of those with bipolar disorder. Patients with schizoaffective disorder predominantly exhibited low to moderate dependence (54.54%), whereas those with paranoid disorder predominantly presented low dependence (75.00%). Patients with epilepsy-related mental disorders had a notably high rate of high and very high dependence (87.50%). Patients with intellectual disability with mental disorders presented diverse levels of dependence, with a predominance of high dependence (31.81%) (**Table 5**).

**Table 5:** Severity of nicotine dependence across mental disorder diagnoses (FTND Criteria).

Dependence level	Schizophrenia (n=217)	Bipolar disorder (n=122)	Schizoaffective disorder (n=11)	Paranoid disorder (n=4)	Epilepsy-related mental disorders (n=8)	Intellectual disability with mental disorders (n=22)
Very low	19 (8.76)	10 (8.20)	1 (9.09)	0 (0.00)	0 (0.00)	3 (13.64)
Low	54 (24.88)	33 (27.05)	4 (36.36)	3 (75.00)	0 (0.00)	5 (22.73)
Moderate	32 (14.75)	19 (15.57)	1 (9.09)	0 (0.00)	1 (12.50)	2 (9.09)
High	78 (35.94)	44 (36.07)	5 (45.46)	1 (25.00)	5 (62.50)	7 (31.81)
Very high	34 (15.67)	16 (13.11)	0 (0.00)	0 (0.00)	2 (25.00)	5 (22.73)

FTND, Fagerström Test for Nicotine Dependence.

## 4 Discussion

### 4.1 Analysis of the current status of tobacco use among patients with SMI

The findings indicated that the prevalence of smoking among patients with SMI was 52.03%, which is higher than the 23.2% prevalence reported for the general Chinese population in 2024 (15). This prevalence also exceeds those reported in other Asian countries. For example, a 2013 study conducted in Japan on individuals with mental disorders found a smoking rate of 17.3% among schizophrenia patients (16). The elevated prevalence of smoking among SMI patients may be attributed to a complex interplay of biological, psychological, and sociological factors unique to their condition. Specifically, this study reveal that the majority of SMI patients use smoking as a primary coping mechanism for stress and emotional regulation. These findings suggest that smoking behavior plays a critical role in alleviating psychological stress in SMI patients. Given that SMI patients are more susceptible to negative emotions such as anxiety and depression, the self-regulatory effects of nicotine are particularly significant in this context (17–19). The pharmacological properties of nicotine may mitigate psychiatric symptoms to some extent (20), thereby contributing to increased tobacco dependence. Understanding this intricate interaction is essential for developing targeted tobacco intervention strategies and improving the overall health of SMI patients. Additionally, some studies have indicated that smoking may alleviate the side effects of antipsychotic medications (21), which could further explain the high smoking rates among SMI patients.

In this study, only 45 patients (6.10% of the total sample) successfully quit smoking, a proportion markedly lower than that reported in the general population (22). This discrepancy is likely attributable to the higher smoking intensity and longer smoking duration among individuals with SMI, leading to greater nicotine dependence and more substantial challenges in quitting. Among current smokers, 35.68% (n=137) expressed an intention to quit, with male patients demonstrating significantly greater willingness than female patients. This gender disparity may be influenced by differing gender role expectations and access to social support networks. Male patients may be more likely to seek cessation resources and support, whereas female patients may face greater barriers in obtaining social support and enhancing self-efficacy (23). Therefore, the specific needs of female patients should be given special consideration when designing and implementing smoking cessation interventions, and more targeted support and intervention strategies should be developed.

The study also revealed that the majority of patients desiring to quit smoking predominantly opted for self-cessation methods, whereas a smaller proportion utilized smoking cessation clinics or other specialized services. This finding indicates that patients with SMIs may have limited access to diverse cessation options and specialized support services. Smoking cessation clinics, as comprehensive interventions, offer tailored counselling, medication, and psychological support, which can significantly increase the success rates of smoking cessation (24). Therefore, it is important to promote the use and accessibility of smoking cessation clinics to increase the effectiveness of smoking cessation in patients with SMI. Future research should explore how to effectively integrate these specialized services into the daily care of patients with SMIs to promote broader behavioral changes towards smoking cessation.

### 4.2 Analysis of factors influencing tobacco use in patients with SMI

This study of 738 patients with SMI identified several risk factors for smoking, including male gender, Han ethnicity, economic hardship, family members smoking, outpatients, a positive family history of mental illness, and history of substance and alcohol use. These findings highlight the multifaceted nature of smoking in this population and inform targeted intervention strategies.

First, the significance of gender differences in smoking behavior among patients with mental illness aligns with findings from previous studies (25). The greater prevalence of smoking among male patients than among female patients may be closely associated with socio-cultural factors (26, 27). In many cultural contexts, smoking among men is seen as a social behavior or stress relief (28). Additionally, sex hormones may also contribute to this disparity (26, 29).

Second, this study revealed that smoking behavior varies significantly across different ethnicities. Specifically, the smoking prevalence among Han Chinese patients was notably greater than that among other ethnic groups. These disparities may be attributed to differences among ethnic groups in terms of cultural customs, lifestyle practices, and the level of awareness regarding the health risks associated with smoking (30). Furthermore, traditional attitudes toward smoking vary across ethnicities, and the prevalent social interaction patterns and cultural norms in Han-majority regions may contribute to greater exposure to and adoption of smoking behaviors (31). Although genetic factors cannot be entirely discounted, the observed ethnic differences likely reflect, at least in part, the influence of cultural background-particularly considering historical data indicating a consistently high smoking rate among the Han Chinese population over an extended period (32).

The impact of economic status on the smoking behavior of people with SMI is also of concern. Economically disadvantaged patients are more likely to engage in smoking as both a coping mechanism for life and a psychiatric stressor and because of their preference for less expensive tobacco products owing to financial constraints (33). Additionally, economic deprivation may restrict their access to health education and cessation support (34–36), thereby exacerbating the persistence of smoking behavior.

The impact of family members’ smoking behavior on patients should not be underestimated. Family members’ smoking not only provides patients with someone to emulate, but also may convey positive messages about smoking through family interactions, ignoring the health risks (37–39). In addition, family members’ smoking may cause patients to be exposed to second-hand smoke, increasing the risk of smoking (40).

It is noteworthy that this study identified a significantly higher prevalence of smoking among outpatients (65.73%) compared to inpatients (46.48%). This discrepancy may be closely linked to the structural characteristics of the medical management system for individuals with mental illness in China. Inpatients generally reside in controlled and supervised environments where tobacco use is strictly regulated, thereby limiting opportunities for smoking (41). In contrast, upon discharge and reintegration into community life, outpatients are more likely to encounter smoking-related triggers within familial or social contexts—such as exposure to household smokers or peer pressure—which can facilitate relapse or sustained tobacco use. Furthermore, outpatient populations frequently confront elevated levels of psychosocial stressors, including occupational instability and societal stigma, which may contribute to the adoption or continuation of smoking as a maladaptive coping mechanism, thereby increasing the likelihood of tobacco dependence (39). These findings underscore the necessity of developing targeted smoking cessation interventions for outpatient populations that integrate robust community-based support systems with tailored modifications to the domestic environment.

Patients with a positive family history of mental illness are at increased risk of smoking, indicating that genetic factors may significantly contribute to smoking behavior (42, 43). Having a mental illness in the family may imply a genetic susceptibility, which not only influences the onset of the illness but also may influence dependence on tobacco (44).

Finally, the associations between a history of drug exposure and alcohol use with smoking behavior have also been well-documented. Drug and alcohol use frequently co-occur with smoking, potentially because their neurobiological interactions that collectively impact the brain’s reward system and stress response (45–47). These maladaptive behaviors reinforce each other, exacerbating the patients’ dependence.

### 4.3 Analysis of nicotine dependence levels in patients with SMI

Our study of 384 smokers with psychosis revealed significant heterogeneity in nicotine dependence across different psychiatric subtypes. Patients with schizophrenia presented the highest level of dependence (51.61%), potentially attributable to dysfunction in the dopaminergic system (48–50). Bipolar disorder patients also showed high dependence (49.18%), which may be linked to neurobiological abnormalities and the mood-stabilizing effects of nicotine (51, 52). Nicotine dependence among individuals with paranoid disorder is relatively uncommon, occurring in only 25.00% of cases. This lower prevalence may be attributed to the unique cognitive structures and behavioral patterns associated with this condition, which may render nicotine a less favorable coping mechanism. Patients with epilepsy-related mental disorders have a high rate of substance dependence (87.50%), likely due to neuroanatomical damage in the prefrontal-limbic system caused by repeated epileptic activity. Frequent seizures may impair frontal cortex function, reducing self-control and increasing vulnerability to nicotine addiction (53, 54). The intellectual disability with mental disorders was highly dependent (31.81%), indicating the need to consider intellectual disability in treatment (55). The results of this research underscore the complexity of nicotine dependence among individuals with SMI, emphasizing the need for specialized interventions that are tailored to specific subtypes of dependence. However, a subgroup analysis of FTND across different diagnostic categories was not feasible due to limited sample sizes within certain diagnostic groups. Consequently, future studies should aim to recruit larger cohorts of individuals with SMI to facilitate more comprehensive and reliable analyses.

### 4.4 Recommendations for clinical practice

Based on the diagnosis-specific dependence patterns, key risk factors, and behavioral characteristics identified in this study, we developed a series of stratified intervention strategies aimed at achieving precise and effective responses. For patients with schizophrenia and bipolar disorder, who exhibit a significantly high rate of nicotine dependence (over 49%), it is recommended that an integrated approach combining pharmacological and behavioral interventions be implemented at the early stage of smoking cessation due to their high-dependence profiles. In contrast, for the group with relatively low dependence—such as approximately 75% of patients with paranoid psychosis—the primary focus should be on enhancing motivation to quit smoking. To facilitate timely identification and intervention, nicotine dependence assessment tools, such as the FTND scale, can be integrated into electronic medical record systems to automatically flag high-risk outpatients and enable intelligent referral to smoking cessation services. To address financial barriers, community health centers should provide free nicotine replacement therapy (NRT), while efforts should also be made to expand health insurance coverage to include non-nicotine medications like varenicline. For female patients, a mutual support platform has been established, and personalized smoking cessation guidance tailored to individual hormonal cycles is offered. Additionally, during home visits, healthcare providers should assess family members’ smoking status and deliver brief 5A interventions to those who smoke, aiming to foster a supportive family environment conducive to smoking cessation and to promote the patient’s recovery.

### 4.5 Limitations and future directions

This study identified factors influencing smoking behavior among patients with SMIs but has several limitations. First, the cross-sectional design prevents causal conclusions; longitudinal studies are needed to confirm the findings. Second, the sample has limited geographical and cultural representation, which may affect the generalizability of the results. Third, reliance on self-reported smoking data could introduce bias; future studies should include objective biomarkers. Fourth, although age and patient status (outpatient/inpatient) were linked to smoking behavior, no stratified analysis was conducted, limiting understanding of how these factors influence different groups. Finally, the study did not evaluate how smoking affects patient outcomes. Future research should involve larger, multi-center samples across regions to better understand the impact of age and treatment setting on smoking. This will help identify risk patterns, guide interventions for different age groups, clarify the influence of environmental and psychological factors, and support more accurate risk assessments and targeted clinical advice.

## 5 Conclusion

In conclusion, this study provides a new perspective for understanding tobacco use behavior and its influencing factors in patients with SMI, and provides a basis for developing effective smoking cessation intervention strategies. Future studies should continue to explore the relevant mechanisms in depth and conduct more extensive intervention studies to improve the health and quality of life of patients with SMI.

## Data Availability

The raw data supporting the conclusions of this article will be made available by the authors, without undue reservation.

## References

[1] Wen H Xie C Shi F Liu Y Liu X Yu C. Trends in deaths attributable to smoking in China, Japan, United Kingdom, and United States from 1990 to 2019. Int J Public Health. (2022) 67:1605147/BIBTEX. doi: 10.3389/IJPH.2022.1605147/BIBTEX, PMID: 36188749 PMC9519860

[2] Zeng Q Zhang C Su F Wan Y Tu WJ Hu H. Prevalence, cessation, and geographical variation of smoking among middle-aged and elderly adults in China: A population-based study. Tob Induc Dis. (2024) 22:133. doi: 10.18332/TID/190247, PMID: 39034965 PMC11259031

[3] Nargis N Faruque GM Ahmed M Huq I Parven R Wadood SN. A comprehensive economic assessment of the health effects of tobacco use and implications for tobacco control in Bangladesh. Tob Control. (2022) 31:723–9. doi: 10.1136/TOBACCOCONTROL-2020-056175, PMID: 33653817

[4] World Health Organization. World mental health report: transforming mental health for all. Geneva (2022). Available at: https://iris.who.int/handle/10665/356119 (Accessed November 10, 2024).

[5] Zhang W Ma N Wang X Wu X Zhao M Chen R. Management and services for psychosis in the People′s Republic of China in 2020. Chin J Psychiatry. (2022) 55:122–8. doi: 10.3760/cma.j.cn113661-20210818-00252

[6] Chen M Huang Y Peng G Yang G Deng W. Analysis of health management effects of type 2 diabetes in schizophrenia patients in Huizhou. Bull Dis Control Prevention (China). (2022) 37:13–7. doi: 10.13215/j.cnki.jbyfkztb.2108024

[7] Li C Yao Y Yang Z. Norms, competence, and support: issues and strategies in the case management of patients with severe mental disorders – A survey based on district H in shanghai city. Soc Work Manage. (2023) 23:56–68. doi: https://shgzygl.gdut.edu.cn/CN/Y2023/V23/I5/56

[8] Fornaro M Carvalho AF De Prisco M Mondin AM Billeci M Selby P. The prevalence, odds, predictors, and management of tobacco use disorder or nicotine dependence among people with severe mental illness: Systematic review and meta-analysis. Neurosci Biobehav Rev. (2022) 132:289–303. doi: 10.1016/j.neubiorev.2021.11.039, PMID: 34838527

[9] Sagud M Mihaljevic Peles A Pivac N. Smoking in schizophrenia: recent findings about an old problem. Curr Opin Psychiatry. (2019) 32:402–8. doi: 10.1097/YCO.0000000000000529, PMID: 31135490

[10] Gilbody S Peckham E Bailey D Arundel C Heron P Crosland S. Smoking cessation for people with severe mental illness (SCIMITAR+): a pragmatic randomised controlled trial. Lancet Psychiatry. (2019) 6:379–90. doi: 10.1016/S2215-0366(19)30047-1, PMID: 30975539 PMC6546931

[11] Rajan S Mitchell A Zavala GA Podmore D Khali H Chowdhury AH. Tobacco use in people with severe mental illness: Findings from a multi-country survey of mental health institutions in South Asia. Tob Induc Dis. (2023) 21:1–13. doi: 10.18332/tid/174361, PMID: 38098747 PMC10720264

[12] Huang Y Liu Z Wang H Guan X Chen H Ma C. The China Mental Health Survey (CMHS): I. background, aims and measures. Soc Psychiatry Psychiatr Epidemiol. (2016) 51:1559–69. doi: 10.1007/S00127-016-1270-Z, PMID: 27796403

[13] Global Adult Tobacco Survey Collaborative Group. Global Adult Tobacco Survey (GATS): Core Questionnaire with Optional Questions. Atlanta, GA (2020).

[14] Pan J Jin W Wang X Bai C. Psychometric property of Chinese version of the Fagerstrom Test of Nicotine Dependence. Chin J Asthma. (2010) 30:266–9. doi: 10.3760/cma.j.issn.1673-436x.2010.05.004

[15] Chinese Center for Disease Control and Prevention. China Adult Tobacco Survey 2024. China: China Adult Tobacco Use Survey Questionnaire (2025).

[16] Umene-Nakano W Yoshimura R Hoshuyama T Yoshii C Hayashi K Nakano H. Current smoking rate in patients with psychiatric disorders in Japan: Questionnaire survey. Psychiatry Res. (2013) 210:268–73. doi: 10.1016/j.psychres.2013.03.024, PMID: 23601794

[17] Lackner CL Thompson B Santesso DL Wade TJ Segalowitz SJ. Perinatal nicotine exposure relates to stimulus-locked event-related potentials in early adolescence during an emotional go/no-go task. Neurotoxicol Teratol. (2023) 97:107175. doi: 10.1016/j.ntt.2023.107175, PMID: 37028464

[18] Szerman N Parro C Vega P Basurte-Villamor I Ruiz-Veguilla M. Tobacco use disorder in patients with other mental disorders: a dual disorder perspective from clinical neuroscience. Front Psychiatry. (2024) 15:1427561. doi: 10.3389/fpsyt.2024.1427561, PMID: 39465048 PMC11502350

[19] Nagawa CS Lane IA McKay CE Kamberi A Shenette LL Kelly MM. Use of a rapid qualitative method to inform the development of a text messaging intervention for people with serious mental illness who smoke: formative research study. JMIR Form Res. (2022) 6:e40907. doi: 10.2196/40907, PMID: 36342765 PMC9679928

[20] Zhang XY Liang J Chen DC Xiu MH He J Cheng W. Cigarette smoking in male patients with chronic schizophrenia in a chinese population: prevalence and relationship to clinical phenotypes. PloS One. (2012) 7:e30937. doi: 10.1371/journal.pone.0030937, PMID: 22347412 PMC3274516

[21] Salokangas RKR Saarijärvi S Taiminen T Lehto H Niemi H Ahola V. Effect of smoking on neuroleptics in schizophrenia. Schizophr Res. (1997) 23:55–60. doi: 10.1016/S0920-9964(96)00083-7, PMID: 9050128

[22] Anthenelli RM Benowitz NL West R St Aubin L McRae T Lawrence D. Neuropsychiatric safety and efficacy of varenicline, bupropion, and nicotine patch in smokers with and without psychiatric disorders (EAGLES): a double-blind, randomised, placebo-controlled clinical trial. Lancet. (2016) 387:2507–20. doi: 10.1016/S0140-6736(16)30272-0, PMID: 27116918

[23] Wang X. Gender-dependent effects and neurobiological mechanisms in nicotine withdrawal syndrome. Adv Psychol. (2021) 11:249–57. doi: 10.12677/AP.2021.111028

[24] Lu C Chen X Li J Wang R Jin X Li H. Review of domestic and international evidence-based smoking cessation guidelines and smoking cessation clinics. Chin J Health Educ. (2024) 40:1003–9. doi: 10.16168/j.cnki.issn.1002-9982.2024.11.009

[25] Martins-da-Silva AS Torales J Becker RFV Moura HF Waisman Campos M Fidalgo TM. Tobacco growing and tobacco use. Int Rev Psychiatry. (2022) 34:51–8. doi: 10.1080/09540261.2022.2034602, PMID: 35584014

[26] Yue Y Hong L Guo L Gao X Deng J Huang J. Gender differences in the association between cigarette smoking, alcohol consumption and depressive symptoms: a cross-sectional study among Chinese adolescents. Sci Rep. (2015) 5:17959. doi: 10.1038/SREP17959, PMID: 26639938 PMC4671152

[27] Verplaetse TL Smith PH Pittman BP Mazure CM McKee SA. Associations of gender, smoking, and stress with transitions in major depression diagnoses. Yale J Biol Med. (2016) 89:123–9., PMID: 27354839 PMC4918874

[28] Kliewer W Lecaj R Wan NM. Cigarette smoking in male high school students in Myanmar: developmental differences in risk and promotive factors. J Child Fam Stud. (2023) 32:1192–203. doi: 10.1007/s10826-022-02340-y

[29] Menson KE Coleman SRM. Smoking and pulmonary health in women: A narrative review and behavioral health perspective. Prev Med (Baltim). (2024) 185:108029. doi: 10.1016/j.ypmed.2024.108029, PMID: 38851402

[30] Wang J Ma H Wang Z Zhang Y. Prevalence of tobacco and alcohol use in ethnic Hui and Han residents in Ningxia. Zhonghua Liu Xing Bing Xue Za Zhi. (2015) 36:1231–5. doi: 10.3760/cma.j.issn.0254-6450.2015.11.010, PMID: 26850242

[31] Liu S Zhou H He W Yang J Yin X Shalayiding S. Risk profiling of tobacco epidemic and estimated number of smokers living in China: a cross-sectional study based on PBICR. BMC Public Health. (2024) 24:2219. doi: 10.1186/S12889-024-18559-X, PMID: 39148035 PMC11325620

[32] Zhang Y Fan X Li S Wang Y Shi S Lu H. Prevalence and risk factors of hypertension among Hui population in China: A systematic review and meta-analysis based on 30,565 study participants. Zhonghua Liu Xing Bing Xue Za Zhi. (2015) 36:1231–35. doi: 10.1097/MD.0000000000025192, PMID: 33950917 PMC8104273

[33] Paul CL Ross S Bryant J Hill W Bonevski B Keevy N. The social context of smoking: A qualitative study comparing smokers of high versus low socioeconomic position. BMC Public Health. (2010) 10:2219. doi: 10.1186/1471-2458-10-211, PMID: 20420707 PMC2868819

[34] Sakuma KLK Pierce JP Fagan P Nguyen-Grozavu FT Leas EC Messer K. Racial/ethnic disparities across indicators of cigarette smoking in the era of increased tobacco control, 1992-2019. Nicotine Tob Res. (2021) 23:909–19. doi: 10.1093/NTR/NTAA231, PMID: 33196799 PMC8522466

[35] Saha SP Bhalla DK Whayne TF Gairola CG. Cigarette smoke and adverse health effects: An overview of research trends and future needs. Int J Angiol. (2007) 16:77–83. doi: 10.1055/S-0031-1278254, PMID: 22477297 PMC2733016

[36] Yang X Galárraga O Cao W Lin H Cao F Chang C. Financial incentive interventions for smoking cessation among Chinese smokers: study protocol for a cluster randomised controlled trial. BMJ Open. (2024) 14:e080344. doi: 10.1136/BMJOPEN-2023-080344, PMID: 38684254 PMC11086505

[37] Wysota CN Topuridze M Sargsyan Z Dekanosidze A Sturua L Kegler MC. Psychosocial factors, smoke-free restrictions, and media exposure in relation to smoking-related attitudes and behaviors among adults in Armenia and Georgia. Int J Environ Res Public Health. (2021) 18:4013. doi: 10.3390/ijerph18084013, PMID: 33920451 PMC8068968

[38] Steeger CM Bailey JA Epstein M Hill KG. The link between parental smoking and youth externalizing behaviors: Effects of smoking, psychosocial factors, and family characteristics. Psychol Addictive Behav. (2019) 33:243–53. doi: 10.1037/adb0000444, PMID: 30667236 PMC6688600

[39] Huddlestone L Shoesmith E Pervin J Lorencatto F Watson J Ratschen E. A systematic review of mental health professionals, patients, and carers’ Perceived barriers and enablers to supporting smoking cessation in mental health settings. Nicotine Tob Res. (2022) 24:945–54. doi: 10.1093/NTR/NTAC004, PMID: 35018458 PMC9199941

[40] Wang MP Ho SY Lo WS Lam TH. Smoking family, secondhand smoke exposure at home, and nicotine addiction among adolescent smokers. Addictive Behav. (2012) 37:743–6. doi: 10.1016/J.ADDBEH.2012.02.016, PMID: 22406053

[41] Shoesmith E Huddlestone L Pervin J Shahab L Coventry P Coleman T. Promoting and maintaining changes in smoking behavior for patients following discharge from a smoke-free mental health inpatient stay: development of a complex intervention using the behavior change wheel. Nicotine Tob Res. (2023) 25:729–37. doi: 10.1093/NTR/NTAC242, PMID: 36250614 PMC10032184

[42] Loukola A Hällfors J Korhonen T Kaprio J. Genetics and smoking. Curr Addict Rep. (2014) 1:75–82. doi: 10.1007/S40429-013-0006-3/METRICS 24778978 PMC4000030

[43] Asharani PV Seet VAL Abdin E Kumar FDS Wang P Roystonn K. Smoking and mental illness: prevalence, patterns and correlates of smoking and smoking cessation among psychiatric patients. Int J Environ Res Public Health. (2020) 17:1–14. doi: 10.3390/IJERPH17155571, PMID: 32752263 PMC7432787

[44] Barkhuizen W Dudbridge F Ronald A. Genetic overlap and causal associations between smoking behaviours and mental health. Sci Rep. (2021) 11:1–13. doi: 10.1038/s41598-021-93962-7, PMID: 34290290 PMC8295327

[45] Martin-Soelch C. Neuroadaptive changes associated with smoking: structural and functional neural changes in nicotine dependence. Brain Sci. (2013) 3:159–76. doi: 10.3390/BRAINSCI3010159, PMID: 24961312 PMC4061825

[46] Verplaetse TL McKee SA. An overview of alcohol and tobacco/nicotine interactions in the human laboratory. Am J Drug Alcohol Abuse. (2017) 43:186–96. doi: 10.1080/00952990.2016.1189927, PMID: 27439453 PMC5588903

[47] Adams S. Psychopharmacology of tobacco and alcohol comorbidity: a review of current evidence. Curr Addict Rep. (2017) 4:25–34. doi: 10.1007/S40429-017-0129-Z, PMID: 28357192 PMC5350203

[48] Barr RS Culhane MA Jubelt LE Mufti RS Dyer MA Weiss AP. The effects of transdermal nicotine on cognition in nonsmokers with schizophrenia and nonpsychiatric controls. Neuropsychopharmacology. (2008) 33:480–90. doi: 10.1038/sj.npp.1301423, PMID: 17443126

[49] Featherstone RE Siegel SJ. The role of nicotine in schizophrenia. Int Rev Neurobiol. (2015) 124:23–78. doi: 10.1016/BS.IRN.2015.07.002, PMID: 26472525

[50] Basu A Ray A. Nicotine dependence and Schizophrenia. Neuropathology of Drug Addictions and Substance Misuse Volume 1: Foundations of Understanding, Tobacco, Alcohol, Cannabinoids and Opioids. (Misuse, UK: Academic Press) (2016). pp. 260–71. doi: 10.1016/B978-0-12-800213-1.00025-0.

[51] Balfour DJK Ridley DL. The effects of nicotine on neural pathways implicated in depression: A factor in nicotine addiction? Pharmacol Biochem Behav. (2000) 66:79–85. doi: 10.1016/S0091-3057(00)00205-7, PMID: 10837846

[52] Heffner JL Strawn JR Delbello MP Strakowski SM Anthenelli RM. The co-occurrence of cigarette smoking and bipolar disorder: phenomenology and treatment considerations. Bipolar Disord. (2011) 13:439–53. doi: 10.1111/J.1399-5618.2011.00943.X, PMID: 22017214 PMC3729285

[53] French JA Kanner AM Bautista J Abou-Khalil B Browne T Harden CL. Efficacy and tolerability of the new antiepileptic drugs II: treatment of refractory epilepsy: report of the Therapeutics and Technology Assessment Subcommittee and Quality Standards Subcommittee of the American Academy of Neurology and the American Epilepsy Society. Neurology. (2004) 62:1261–73. doi: 10.1212/01.WNL.0000123695.22623.32, PMID: 15111660

[54] Gehris M Ijaz A Chakraborty A Jebai R Li W Osibogun O. Epilepsy and nicotine use: Exploring disparities in ENDS and cigarette use among US adults with epilepsy. Epilepsy Behav. (2025) 162:110177. doi: 10.1016/J.YEBEH.2024.110177, PMID: 39612631 PMC11634633

[55] Bhattacharya A Chatterjee S Bhattacharya S Chakraborty R Dan A Singh OP. Brain CT scan study of mentally retarded individuals with comorbid psychiatric disorder. Indian J Public Health Res Dev. (2014) 5:117–21. doi: 10.5958/J.0976-5506.5.1.028

